# Thinking outside the box

**DOI:** 10.1007/s40037-013-0065-0

**Published:** 2013-06-29

**Authors:** G. Peeraer

**Affiliations:** University of Antwerp, Antwerp, Belgium

‘*The real act of discovery consists not in finding new lands, but in seeing with new eyes*’ (Marcel Proust, 1871–1922) [1].

Health care education is developing at a high pace, all around the world. New ideas are needed, new ideas that work and help us meet the goals we set for improving health care education. PME wants to contribute to seeing with new eyes by offering its readers articles that are inspirational by nature. Articles with new ideas or with research based on new ideas that work. This inspiration combined with strong research is what PME is all about.

The author of the first original article of this issue, Sonya Miller, offers us a curriculum focused on informed empathy [2]. As 20 % of the US population is disabled, health care providers are challenged by the demands of disabled patients. They want to be treated as equals, but say that they are often treated unequally. In order to train health care workers’ empathy, a new curriculum was created. The developers thought out of the box in designing this curriculum: as well as involving real (disabled) patients, they also stimulated their students in providing artistic interpretation (poems, photos,…) to effectively develop and enhance informed empathy. In this article, the author presents a new idea that works, underlining this by stating ‘health is influenced by more than a diagnosis, disability, medical professional or hospital’.

In the second original article, Dijksterhuis et al. [3] present the questionnaire they developed which contains questions on the progress test that is used in postgraduate obstetrics and gynaecology in the Netherlands. They asked both educational supervisors and trainees to reflect on acceptability and educational impact. All trainees have to take the test at regular intervals, but failing the test does not have any consequences for the trainees progress.

As postgraduate programmes worldwide are changing from a more loosely structured to a strong assessment culture, thinking outside the box is needed. To be able to make effective changes in this culture, new ideas are called for. The authors prefer assessment that is firmly embedded in the postgraduate training programme over assessment with limited educational effect. Through their research they show that the current system of progress testing is not achieving what should be achieved: helping trainees develop their knowledge up to strong standards (instead of relying too much on sources like the internet). Giving no consequences to failing the test is keeping to tradition. As long as our pattern-seeking brain sends us off on old paths we will not change the current postgraduate learning culture into the learning culture most of us aim for.

At the Cleveland Clinic, ‘clear communication with medical trainees’ was one of the main challenges. Although they considered emails an effective way to communicate with the Clinic, trainees felt overloaded by emails. Therefore the communication team (with authors Greenwald and Stoller) looked at the existing system with new eyes instead of keeping to old patterns; they thought out of their box and developed new systems: a web-based intranet site and an electronic newsletter. The site is updated daily with customized, focused content for its specific audience and it is now the single repository of information relevant for trainees, programme directors and coordinators [4].

The short communications of this issue are inspirational as well: Borges et al. [5] interviewed women with varying cultural and training backgrounds to find out their perspectives on choosing an academic career. Tanck et al. [6] invented the TOPday: a two opportunities of practice day for students in biomedical sciences. Gaughf et al. [7] explored faculty and students’ perceptions of available academic counselling services at an academic health science centre.

The eye opener explains the phenomenon of Pommy medical officers: UK medical doctors working in Australia. The author, Alexander Armstrong, had the feeling that his clinical training in the UK had not prepared him sufficiently for the very competitive process of applying for the speciality of surgery. He therefore decided to literally cross borders and migrate to Australia [8].

As Edward De Bono (1933) says: ‘There is nothing more exciting…than thinking of a new idea. There is nothing more rewarding… than seeing a new idea work. There is nothing more useful… than a new idea that helps you meet a goal.’

This is part of what PME aims for: getting its readers excited and helping them find new ideas that help them meet their goals [9].
